# Quantifying the spreading resistance of an anisotropic thin film conductor

**DOI:** 10.1038/s41598-020-66739-7

**Published:** 2020-06-30

**Authors:** Kazuhiko Seki, Toshitaka Kubo, Nan Ye, Tetsuo Shimizu

**Affiliations:** 1 National Institute of Advanced Industrial Science and Technology (AIST), AIST Tsukuba Central 5, Higashi 1-1-1, Tsukuba, Ibaraki 305-8565 Japan; 2 Yazaki Corporation 1500 Mishuku, Susono-city, Shizuoka 410-1194 Japan

**Keywords:** Electrical and electronic engineering, Applied physics, Graphene, Nanoscale devices

## Abstract

Recently, highly anisotropic conductors, such as multilayer graphene, have been attracting much attention. The local resistivity can be determined by measuring the contact resistance; however, the theoretical expressions of contact resistance have been developed for isotropic slabs but have not been well developed for highly anisotropic film conductors. We obtain theoretical expressions of the spreading resistance below the circular contact for a highly anisotropic film on a bulk slab. The film spreading resistance of isotropic conductors deviates from the bulk spreading resistance when the film thickness is smaller than the contact radius. Nevertheless, the spreading resistance of anisotropic conducting films can be approximated by that of the bulk slabs even when the film thickness is smaller than the contact radius if the in-plane electrical conductivity is larger than the out-of-plane electrical conductivity. Owing to the high in-plane conductivity, the spreading resistance of anisotropic bulk conductors can be lowered from that predicted by the Holm’s equation obtained using the out-of-plane conductivity and the contact radius. We show that these characteristics are beneficial to use the highly anisotropic film as a cover layer when the in-plane conductivity of the film is high and the conductivity of the base slab is low.

## Introduction

Recently, coating by multilayer graphene is receiving increasing attention owing to its high electrical conductivity, resistance to corrosion, low friction, and optical transparency^1–8^. Though the in-plane electrical conductivity of graphene is high, the out-of-plane electrical conductivity is low; hence, the electrical conductivity is highly anisotropic. The local resistivity can be determined by measuring the contact resistance. The electrical contact resistance between a disc electrode and a conductor slab has been attracting attention for many years. For an infinite isotropic conductor, the theoretical ideal resistance in the conductor directly under the contact is known as the spreading resistance and is expressed by Holm’s equation^9^, $R=1/(4a\sigma )$, where *R* represents the spreading resistance, *a* is the radius of the disc electrode and *σ* is the isotropic electrical conductivity of the conductor slab. For anisotropic materials, such as multi-graphene, Holm’s equation should be amended to relate the local resistance and the anisotropic electrical conductivities. The spreading resistance for anisotropic electrical conductors has been developed for bulk slabs but not thoroughly studied for thin films^10,11^. In this paper, we derive the spreading resistance for anisotropic electrical conducting films. We then show that multi-layer graphene could be beneficial for collecting spreading currents below the multi-layer graphene under a certain condition. Anisotropy in electrical conductivity is common in other materials such as nano-composites, layered chalcogenides, and perovskites^12–18^. Some of the results in this paper may also applicable to systems other than multi-layer graphene.

## Spreading resistance of anisotropic conductor

**Figure 1:**
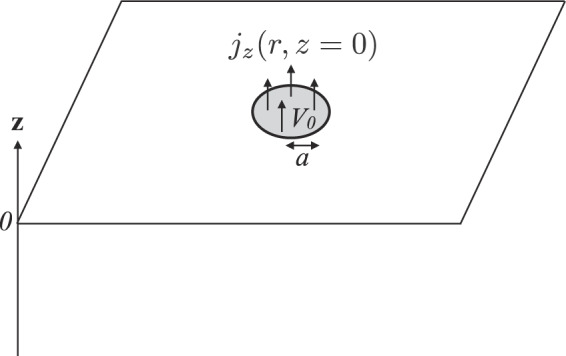
Schematic of a circular contact to a conductor.

As shown in Fig. 1, we consider the spreading resistance for the current passing through the circular domain on an anisotropic conductor. With this setting, the spreading resistance has been investigated experimentally^10,11^. We first give a brief derivation of the spreading resistance for an anisotropic bulk conductors denoted by ${R}_{\infty }$. Then, the results are generalized for thin films and cover layers. The spreading resistance is denoted by *R*_0_ when the film thickness is smaller than any other length scales of interest. We show that the spreading resistance of the cover layer can be lowered from the spreading resistance of the bulk base slab alone denoted by *R*_*b*_ under some conditions.

The contact radius is denoted by *a*. The electrostatic potential at the circular domain is denoted by *V*_0_. The electrical conductivity in the direction parallel to the surface of the circular domain is denoted by ${\sigma }_{\parallel }$ and that in the direction perpendicular to the surface is denoted by ${\sigma }_{\perp }$. The z-axis is defined in the direction perpendicular to the surface and the circular domain surface is located at $z=0$, and the anisotropic conductor is located at the negative values of the $z$-coordinate. We introduce $x$ and $y$ coordinates in the direction parallel to the surface. The current in the anisotropic conductor is denoted by $\overrightarrow{j}$. The current obeys the continuity equation in the steady state, $0={\rm{div}}\,\overrightarrow{j},$ and Ohm’s law ${\overrightarrow{j}}_{x}={\sigma }_{\parallel }{\overrightarrow{E}}_{x}$, ${\overrightarrow{j}}_{y}={\sigma }_{\parallel }{\overrightarrow{E}}_{y}$ and ${\overrightarrow{j}}_{z}={\sigma }_{\perp }{\overrightarrow{E}}_{z}$, where the electric field is related to the electrostatic potential $V$ by $\overrightarrow{E}=-\,{\rm{grad}}\,V$. By using the above equations, we can show that *V* satisfies the anisotropic Laplace equation given by

1 $${\sigma }_{\parallel }{\nabla }_{\parallel }^{2}V+{\sigma }_{\perp }{\nabla }_{\perp }^{2}V=0,$$

where we define ${\nabla }_{\parallel }^{2}={\partial }^{2}/(\partial {x}^{2})+{\partial }^{2}/(\partial {y}^{2})$ and ${\nabla }_{\perp }^{2}={\partial }^{2}/\partial {z}^{2}$. A similar potential function has been used to obtain electric voltage change but not to calculate the spreading resistance^12–16^.

The electric potential inside the circular domain of radius $a$ is *V*_0_ and the potential far below $z=0$ is set to 0^19^. Another boundary condition is that the current outside the circular domain is zero. The solution can be expressed as

2 $$V(r,z)=\frac{2{V}_{0}}{\pi }\,{\int }_{0}^{\infty }\,\frac{dk}{k}\,\sin (ka){J}_{0}(kr)\,\exp \,(kz\sqrt{{\sigma }_{\parallel }/{\sigma }_{\perp }}),$$

where $R$ denotes the distance from the center of the circular domain at $z=0$ and ${J}_{m}(z)$ indicates the Bessel function of the first kind^20^. One can confirm that the boundary conditions are satisfied by using^19,20^.

3 $$(2{V}_{0}/\pi )\,{\int }_{0}^{{\rm{\infty }}}\,\frac{dk}{k}\,\sin (ka){J}_{0}(kr)={V}_{0}\,{\rm{i}}{\rm{f}}\,0\le r\le a\,{\rm{a}}{\rm{n}}{\rm{d}}\,0 < a$$

4 $${\int }_{0}^{\infty }\,\sin (ka){J}_{0}(kr)dk=0\,{\rm{if}}\,r > a.$$

The z-component of the electric field at $z=0$ is obtained from ${E}_{z}(r,z)=-\,{\nabla }_{z}V(r,z)$ as

5 $${E}_{z}(r,z=0)=-\,\frac{2{V}_{0}}{\pi }\sqrt{\frac{{\sigma }_{\parallel }}{{\sigma }_{\perp }}}\,{\int }_{0}^{\infty }\,dk\,\sin (ka){J}_{0}(kr).$$

The current flowing through the circular disk in the z-direction is given by ${j}_{z}(r,z=0)={\sigma }_{\perp }{E}_{z}(r,z=0)$. We can calculate the total current by ${j}_{T}=2\pi \,{\int }_{0}^{a}rdr{j}_{z}(r,z=0)$ using ${\int }_{0}^{a}dr\,r{J}_{0}(kr)=(a/k){J}_{1}(ka)$ and ${\int }_{0}^{\infty }(dk/k)\,\sin (ka){J}_{1}(ka)=1$^20^. The spreading resistance is obtained from $-\,{V}_{0}=R{j}_{T}$ as^10,11^

6 $${R}_{\infty }=\frac{1}{4{\sigma }_{\perp }{a}_{{\rm{eff}}}}=\frac{1}{4a\sqrt{{\sigma }_{\perp }{\sigma }_{\parallel }}},$$

where we defined the effective radius of the circular spot by^10,11^

7 $${a}_{{\rm{eff}}}=a\sqrt{{\sigma }_{\parallel }/{\sigma }_{\perp }}.$$

Equations (6) and (7) are known and have been thoroughly studied experimentally^10,11^. These will be further generalized below for thin films and cover layers.

## Spreading resistance of anisotropic thin layer on an equipotential surface

**Figure 2:**
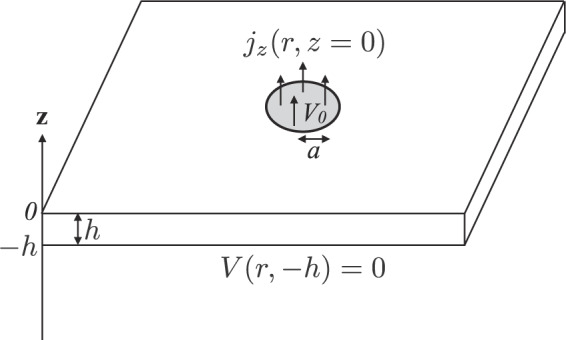
Schematic of a circular contact to a conductor of thickness *h*.

To investigate spreading resistance of an anisotropic thin layer, we consider a circular contact to a thin layer conductor of thickness *h* (see Fig. 2). One side faces the circular contact and the other side faces a metal conductor. For the metal contact with a high electrical conductivity (an equipotential contact interface), we assume a constant potential. Because we are interested in the electrostatic potential difference between the circular contact and the other side, the electrostatic potential inside the circular disk is held at a constant *V*_0_ and that at the opposite side is held at zero potential, which can be expressed as $V(r,-\,h)=0$^21^. The electrostatic potential can be expressed as

8 $$V(r,z)={\int }_{0}^{\infty }\,\frac{dk}{k}{C}_{1}(k){J}_{0}(kr)\left[\exp \left(kz\sqrt{\frac{{\sigma }_{\parallel }}{{\sigma }_{\perp }}}\right)-\exp \left(\,-\,k(z+2h)\sqrt{\frac{{\sigma }_{\parallel }}{{\sigma }_{\perp }}}\right)\right],$$

where ${C}_{1}(k)$ is determined from the boundary conditions given by

9 $$V(r,0)={V}_{0}\,{\rm{if}}\,r\le a$$

10 $${j}_{z}(r,z=0)=0\,{\rm{if}}\,r > a,$$

where the current flowing through the circular disk in the z-direction is given by ${j}_{z}(r,z=0)=\sigma {E}_{z}(r,z=0)$ and ${E}_{z}(r,z)=-\,{\nabla }_{z}V(r,z)$. ${C}_{1}(k)$ can be approximately obtained using Eqs. (3) and (9) as

11 $${C}_{1}(k)=\frac{(2{V}_{0}/\pi )\,\sin (ka)}{1-\exp (\,-\,2kh\sqrt{{\sigma }_{\parallel }/{\sigma }_{\perp }})}.$$

**Figure 3:**
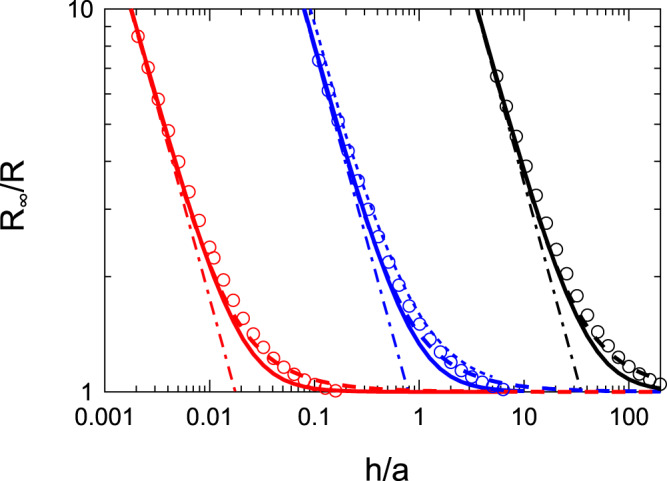
*R*_∞_/*R* plotted as a function of *h*/*a*. The solid lines are obtained using $R$ given by Eq. (13). The dashed-dotted lines are obtained using Eq. (14). The short dashed lines are obtained using Eq. (15). The lower (red) lines indicate the case when ${\sigma }_{\perp }/{\sigma }_{\parallel }=0.0005$. The middle (blue) lines indicate the isotropic case of ${\sigma }_{\parallel }={\sigma }_{\perp }$. The higher (black) lines indicate the case when ${\sigma }_{\perp }/{\sigma }_{\parallel }=2000$. The dots for the case of ${\sigma }_{\parallel }={\sigma }_{\perp }$ indicate the results calculated by the finite element method^24^. The open circles indicate the numerically exact results obtained by the method described in the Appendix A.

Strictly speaking, ${C}_{1}(k)$ does not satisfy Eq. (4) if *h* is kept finite^22–24^. We will show later that the deviation from the exact result is within a few percent. We also show the numerical result which satisfies both boundary conditions given by Eqs. (9) and (10); the improvement is within a few percent. The final result requires numerically solving an integral equation (see also Fig. 3) which satisfies both boundary conditions given by Eqs. (9) and (10); the derivation of the integral equation is shown in the Appendix A. The derivation is based on a method of solving mixed boundary value problems^25,26^.

The z-component of the electric field is given by

12 $${E}_{z}(r,0)=-\,\frac{2{V}_{0}}{\pi }\sqrt{\frac{{\sigma }_{\parallel }}{{\sigma }_{\perp }}}\,{\int }_{0}^{\infty }\,dk\,\sin (ka){J}_{0}(kr)\,\coth (\sqrt{{\sigma }_{\parallel }/{\sigma }_{\perp }}kh)$$

and the spreading resistance can be obtained as

13 $$R=\frac{1}{4a\sqrt{{\sigma }_{\perp }{\sigma }_{\parallel }}}{\left[{\int }_{0}^{\infty }\frac{d\lambda }{\lambda }\sin (\lambda ){J}_{1}(\lambda )\coth (\lambda (h/a)\sqrt{{\sigma }_{\parallel }/{\sigma }_{\perp }})\right]}^{-1}.$$

In the limit of $(h/a)\sqrt{{\sigma }_{\parallel }/{\sigma }_{\perp }} > 1$, Eq. (13) reproduces the exact result for an infinite depth of anisotropic conductors given by Eq. (6). In the opposite limit of $(h/a)\sqrt{{\sigma }_{\parallel }/{\sigma }_{\perp }} < 1$, Eq. (13) reduces to the reasonable limiting expression given by

14 $${R}_{0}=h/({\sigma }_{\perp }\pi {a}^{2}),$$

where we have used ${\int }_{0}^{\infty }d\lambda \,\sin (\lambda ){J}_{1}(\lambda )/{\lambda }^{2}=\pi /4$^20^. *R*_0_ represents the resistance of the cylinder with radius $a$ and length *h*. In this limit, the current spreading beyond the circular edge can be ignored. Equation (13) interpolates between the correct limiting expressions. For isotropic systems, Eq. (13) reduces to the approximate result derived previously^27–29^.

The commonly used empirical equation of the spreading resistance for isotropic materials with the electrical conductivity *σ* is given by $R=\arctan (2h/a)/(2\pi \sigma a)$^30^. Because the anisotropic Laplace equation given by Eq. (1) can be transformed to the isotropic Laplace equation by rescaling *x* with $x/\sqrt{{\sigma }_{\parallel }}$, *y* with $y/\sqrt{{\sigma }_{\parallel }}$, and *z* with $z/\sqrt{{\sigma }_{\perp }}$, *h*/*a* in the expression of $R$ should be transformed into $(h/a)\sqrt{{\sigma }_{\parallel }/{\sigma }_{\perp }}$ for anisotropic conductors. In the limit of $h\to \infty$, the expression of $R$ should reproduce Eq. (6) using $\arctan (x)\to \pi /2$ ($x\to \infty$). Therefore, the empirical equation should be generalized to

15 $$R=\frac{1}{2\pi a\sqrt{{\sigma }_{\parallel }{\sigma }_{\perp }}}\,\arctan \left(\frac{2h}{a}\sqrt{\frac{{\sigma }_{\parallel }}{{\sigma }_{\perp }}}\right),$$

when the electrical conductivities are anisotropic.

In Fig. 3, we show *R*_∞_/*R* as a function of *h*/*a*, where *R*_∞_ is defined by Eq. (6). As explained above, *R*_∞_/*R* of an anisotropic material can be obtained from that of an isotropic material by replacing *h*/*a* with $(h/a)\sqrt{{\sigma }_{\parallel }/{\sigma }_{\perp }}$. The accuracy of the approximate expression given by Eq. (13) and the empirical expression given by Eq. (15) can be examined by comparison with *R*_∞_/*R* obtained from the finite element method for isotropic materials^24^. Small deviation from the numerical result can be observed between *h*/*a* ~ 0.5 and 3 but the errors are within a few percent. In the same figure, we also show the results of Eq. (14). Equation (14) represents the result when the current spreading beyond the circular edge can be ignored. Judging from Fig. 3, the contact resistance is affected by the currents spreading beyond the circular edge when $5 > (h/a)\sqrt{{\sigma }_{\parallel }/{\sigma }_{\perp }} > 0.5$. The same figure also shows that the spreading resistance can be approximately expressed by Eq. (6) when $(h/a)\sqrt{{\sigma }_{\parallel }/{\sigma }_{\perp }} > 5$.

**Figure 4:**
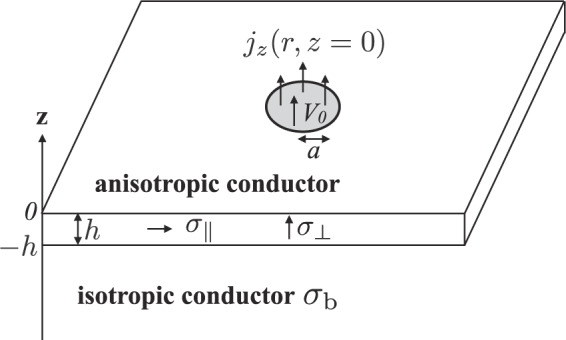
Schematic of a circular contact to a conductor of thickness *h* above the base slab with the electrical conductivity *σ*_*b*_.

According to the material parameters of multilayer graphene, we have ${\sigma }_{\perp }/{\sigma }_{\parallel }=0.0005$ and the current spreads if $h/a > 0.01$ holds. The spreading resistance can be approximately expressed by *R*_∞_ given by Eq. (6) when $h/a > 0.1$. In the opposite limit of ${\sigma }_{\perp }/{\sigma }_{\parallel }=2000$, the current spreads when $h/a > 23$; the spreading resistance is approximately given by Eq. (14) when $h/a < 23$. In both limits, the overall dependence of the spreading resistance on $h/a$ can be obtained from Eq. (15) when the bottom surface of the layer is equipotential. The importance of the bottom boundary condition to the value of the spreading resistance of thin films has been pointed out by studying theoretically an isotropic conducting layer of finite width on an insulating base slab, where currents injected from the sides of the layer flow parallel to the bottom surface^31–33^. In our case, components of electrical currents flowing parallel to the bottom surface increase by lowering the electrical conductivity of the base slab shown in Fig. 4. Below, we study the effect of the electrical conductivity of the base slab on the spreading resistance of the anisotropic conducting layer.

## Spreading resistance of anisotropic cover layer

A possible application of a thin anisotropic layer is as a cover layer for collecting the spreading currents below the cover layer. If the base slab under the cover layer is isotropic and the spreading resistivity is large for this material, the spreading resistivity can be lowered by inserting a thin layer with a high in-plane electrical conductivity between the circular contact and the base slab to collect the spreading currents in the base slab. The overall resistivity can be lowered by covering the anisotropic conductor with a high in-plane electrical conductivity by collecting currents below the cover layer, though the series resistance caused by inserting the thin layer should be taken into account with care. We estimate the gaining condition of the spreading resistance by inserting an anisotropic conductor arising from increasing the effective area despite the increase in the series resistance. First, we show a hand-waving argument. Later, we give a more precise derivation on the condition.

As shown in Fig. 4, we consider a circular contact to a conductor of thickness *h* above the base slab with the electrical conductivity *σ*_*b*_. When ${\sigma }_{\parallel }\gg {\sigma }_{\perp }$ holds, the spreading resistance can be approximated by *R*_∞_ defined by Eq. (6) as long as $h$ exceeds $a\sqrt{{\sigma }_{\perp }/{\sigma }_{\parallel }}$, which is much smaller than *a*. In this case, the spreading resistance in the cover layer can be approximately given by $R=1/(4{a}_{{\rm{eff}}}{\sigma }_{\perp })$. If the spreading resistance is reduced by inserting the cover layer, the gaining condition of the spreading resistance by inserting the cover layer can be approximately expressed as $1/(4a{\sigma }_{b}) > 1/(4{a}_{{\rm{effb}}}{\sigma }_{b})+1/(4{a}_{{\rm{eff}}}{\sigma }_{\perp })$, where *a*_eff_ is defined by Eq. (7) and *a*_effb_ indicates the effective contact radius of the base slab, which could be larger than *a*_eff_ owing to a spreading of the currents in the cover layer. The above inequality holds, if ${a}_{{\rm{effb}}} > {a}_{{\rm{eff}}}$ and $1/(4a{\sigma }_{b}) > 1/(4{a}_{{\rm{eff}}}{\sigma }_{b})+1/(4{a}_{{\rm{eff}}}{\sigma }_{\perp })$ hold. By rearrangement, we obtain ${\sigma }_{b} < \sqrt{{\sigma }_{\parallel }{\sigma }_{\perp }}-{\sigma }_{\perp }$ and

16 $${\sigma }_{b} < \sqrt{{\sigma }_{\parallel }{\sigma }_{\perp }}$$

in the limit of ${\sigma }_{\parallel }\gg {\sigma }_{\perp }$. Equation (16) is the condition that the spreading resistance of the slab base can be lowered by the cover layer for collecting spreading currents, though the insertion of the cover layer may be regarded as adding series resistance. The effective spreading resistance is decreased by the large effective radius of the contact area of the anisotropic conductor with a high parallel conductivity.

The more precise condition can be derived as shown in the Appendix B^34,35^. The spreading resistance is approximately obtained as [See the Appendix B for the derivation].

17 $$R=\frac{1}{4a}\frac{1}{\sqrt{{\sigma }_{\perp }{\sigma }_{\parallel }}}\frac{1}{{C}_{F}},$$

and the inverse correction factor is given by

18 $${C}_{F}={\int }_{0}^{\infty }\,\frac{d\lambda }{\lambda }\,\sin (\lambda ){J}_{1}(\lambda )\frac{\sinh [\sqrt{{\sigma }_{\parallel }/{\sigma }_{\perp }}(h/a)\lambda ]+({\sigma }_{b}/\sqrt{{\sigma }_{\parallel }{\sigma }_{\perp }})\,\cosh [\sqrt{{\sigma }_{\parallel }/{\sigma }_{\perp }}(h/a)\lambda ]}{\cosh [\sqrt{{\sigma }_{\parallel }/{\sigma }_{\perp }}(h/a)\lambda ]+({\sigma }_{b}/\sqrt{{\sigma }_{\parallel }{\sigma }_{\perp }})\,\sinh [\sqrt{{\sigma }_{\parallel }/{\sigma }_{\perp }}(h/a)\lambda ]}.$$

**Figure 5:**
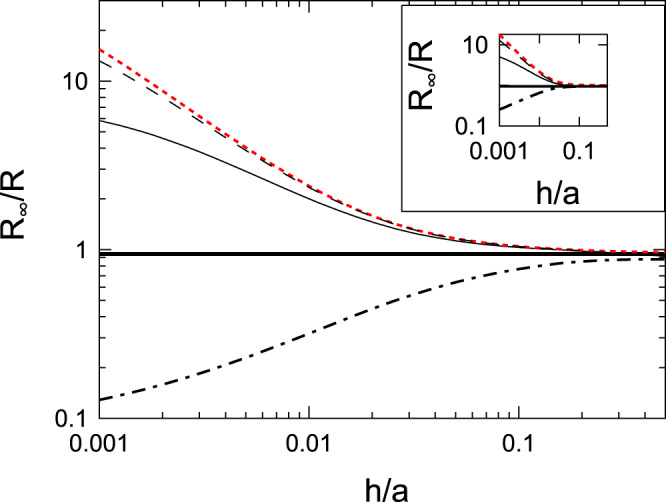
*R*_∞_/*R* plotted as a function of *h*/*a* when ${\sigma }_{\perp }/{\sigma }_{\parallel }=0.0005$. The red dots indicate the limiting case of the equipotential interface below a conductive layer. The dashed line, thin solid line, thick solid line and dashed-dotted line indicate the results of ${\sigma }_{b}/\sqrt{{\sigma }_{\parallel }{\sigma }_{\perp }}=100$, 10, 1, and 0.1, respectively. These lines are obtained using the numerical exact method shown in the Appendix B below Eq. (64). The insert shows the corresponding approximate results, where *R* is obtained from Eq. (17) with the inverse correction factor given by Eq. (18) for the black lines and by Eq. (13) for the red dots.

For isotropic systems, Eqs. (17) and (18) reduce to the approximate expression obtained from the relation between the current and the applied potential in^27^. In the limit of $h\to \infty$, Eq. (18) reproduces *R*_∞_ given by Eq. (6). In the opposite limit of $h\to 0$, Eq. (18) reduces to ${R}_{{\rm{b}}}=1/(4a{\sigma }_{{\rm{b}}})$, which is the spreading resistance of the base slab below the cover layer. In the limit of ${\sigma }_{b}\to \infty$, Eq. (18) reproduces the spreading resistance of the layer on a highly conductive metal slab (an equipotential contact interface) given by Eq. (13). In the limit of ${\sigma }_{b}\to 0$, Eq. (18) should represent the spreading resistance of a layer on an insulator. Although we obtain

19 $${C}_{F}={\int }_{0}^{\infty }\,\frac{d\lambda }{\lambda }\,\sin (\lambda ){J}_{1}(\lambda )\,\tanh [\sqrt{{\sigma }_{\parallel }/{\sigma }_{\perp }}(h/a)\lambda ]$$

in this limit, the larger error is found when $h/a\ll 1$ compared to the case of the cover layer on a metal as indicated in Fig. 5. When the cover layer is on an insulator, the method described in the Appendix B below Eq. (64) is required to take into account the spreading currents flow in the horizontal direction; in this limit, the electrical currents flow into the layer from the horizontal direction and the current is non-zero despite the absence of vertical electrical currents from the insulator base slab. By comparing Eqs. (13) to (17) with Eq. (19), the spreading resistance of the thin layer is increased on the insulator substitute compared with that on the conductor. If we take the limit of $h\to 0$ in Eq. (19), *R* given by Eq. (17) diverges. The reason is that the current flow above the base slab is parallel to the interface between the cover layer and the base slab, hence the cross section area of the current flow tends to vanish as $h\to 0$^36^.

In Fig. 5, we show the inverse correction factor as a function of $h/a$ calculated using the numerically exact method described in the Appendix B below Eq. (64). The red dots indicate the limit of the highly conductive base slab (an equipotential contact interface). The qualitative features are preserved by the corresponding approximate results obtained from Eq. (18) shown in the inset. By decreasing the values of *σ*_*b*_, the inverse correction factor decreases when $(h/a)\sqrt{{\sigma }_{\parallel }/{\sigma }_{\perp }} < 1$ holds. When ${\sigma }_{b}/\,\sqrt{{\sigma }_{\parallel }{\sigma }_{\perp }}=1$, the inverse correction factor is close to one and the resistance is virtually equal to the case of $h\to \infty$. By further decreasing the values of *σ*_*b*_, the inverse correction factor decreases below 1 when $(h/a)\sqrt{{\sigma }_{\parallel }/{\sigma }_{\perp }} < 1$ holds.

**Figure 6:**
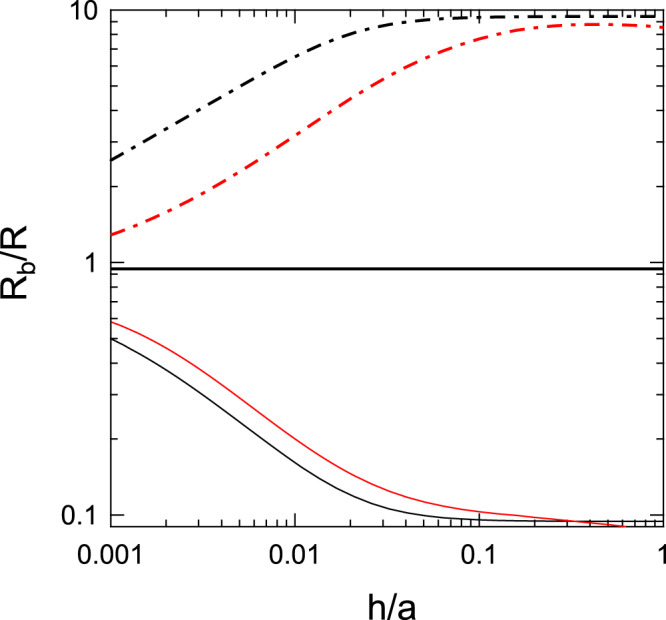
*R*_*b*_/*R* plotted as a function of *h*/*a* when ${\sigma }_{\perp }/{\sigma }_{\parallel }=0.0005$. ${R}_{{\rm{b}}}=1/(4a{\sigma }_{{\rm{b}}})$ and indicates the spreading resistance of the base slab alone. The (black) dashed-dotted line, the (black) thick solid line and the (black) thin solid line indicate ${\sigma }_{b}/\sqrt{{\sigma }_{\parallel }{\sigma }_{\perp }}=0.1$, 1, and 10, respectively. These lines are obtained using *R* with the inverse correction factor given by Eq. (18). Numerically exact results obtained by the method described in the Appendix B below Eq. (64) are shown by the corresponding lines in red. [The results for ${\sigma }_{b}/\sqrt{{\sigma }_{\parallel }{\sigma }_{\perp }}=1$ overlap with the approximate values and are not shown in red].

In Fig. 6, we show *R*_*b*_/*R* as a function of $h/a$ when ${\sigma }_{\perp }/{\sigma }_{\parallel }=0.0005$. The ratio is larger than $1$ when ${\sigma }_{b}/\,\sqrt{{\sigma }_{\parallel }{\sigma }_{\perp }}=0.1$. The results indicate that the conductance is increased by inserting the cover layer. The ratio given by ${R}_{b}/R$ increases by increasing $h/a$ and saturates when $h/a$ is increased over the threshold value given by $h/a=\sqrt{{\sigma }_{\perp }/{\sigma }_{\parallel }}$. In the opposite case, when ${\sigma }_{b}/\,\sqrt{{\sigma }_{\parallel }{\sigma }_{\perp }}=10$, the conductance is decreased by inserting the cover layer. The ratio given by ${R}_{b}/R$ decreases by increasing $h/a$ and saturates again when $h/a$ is increased over the threshold value given by $h/a=\sqrt{{\sigma }_{\perp }/{\sigma }_{\parallel }}$. When ${\sigma }_{b}/\,\sqrt{{\sigma }_{\parallel }{\sigma }_{\perp }}=1$, *R*_*b*_/*R* is virtually independent of the values of *h*/*a*; the spreading resistance is not influenced by a cover layer of any thickness. These results obtained using Eq. (18) are further corroborated by using the numerically exact results obtained from the method described in the Appendix B below Eq. (64); the qualitative feature is not altered. However, a quantitative deviation can be seen when ${\sigma }_{b}/\,\sqrt{{\sigma }_{\parallel }{\sigma }_{\perp }} < 1$ and $(h/a)\sqrt{{\sigma }_{\parallel }/{\sigma }_{\perp }} < 1$. When ${\sigma }_{b}/\,\sqrt{{\sigma }_{\parallel }{\sigma }_{\perp }} < 1$, the direction of the electric field in the anisotropic layer just above the base slab tends to be parallel to the interface surface and still influences the field direction around the circular contact region if $(h/a)\sqrt{{\sigma }_{\parallel }/{\sigma }_{\perp }} < 1$ holds. In the approximate expression given by Eq. (18), such an effect is not fully taken into account because the boundary condition imposed for the current at the circular contact region is not satisfied. By applying the numerical method described in the Appendix B below Eq. (64), the change in the field lines can be followed accurately.

In Fig. 6, ${R}_{b}/R > 1$ if the spreading resistance on the cover layer is lower than the spreading resistance of the base slab alone. The red dashed-dotted line indicates ${R}_{b}/R > 1$ when ${\sigma }_{b}/\,\sqrt{{\sigma }_{\parallel }{\sigma }_{\perp }}=0.1$; *R* decreases by the presence of the top layer and by increasing the layer thickness denoted by *h* when the electrical conductivity of the base slab is low. The results correlate well with experimental findings, where graphene layers are immobilized by polyaminophenylene (PAP) and the contact resistance is measured by conducting prove atomic force microscopy (CP-AFM)^37^. Though PAP is electrically resistive, the contact resistance values are lowered by the presence of graphene layers and smaller for the larger number of graphene layers.

## Conclusion

As a highly anisotropic conductor, we consider multi-layer graphene. For a given sheet resistance (*R*_□_) and vertical distance between layers (*d*_*s*_), the parallel (in-plane) electrical conductivity can be obtained from ${\sigma }_{\parallel }=1$/(*R*_□_*d*_*s*_). However, the vertical (out of plane) electrical conductivity can be directly obtained from the vertical resistivity (${\rho }_{\perp }$) as ${\sigma }_{\parallel }=1/{\rho }_{\perp }$. By substituting the parameter values of graphene (*R*_□_ = 500 Ω_□_, ${d}_{s}=0.3\,{\rm{nm}}$, ${\rho }_{\perp }=3\times {10}^{4}$ *μ*Ωcm)^38–40^, we find $\sqrt{{\sigma }_{\parallel }{\sigma }_{\perp }}=1/\sqrt{{R}_{\square }{d}_{s}{\rho }_{\perp }}\approx 1.5\times {10}^{3}$ S/cm and ${\sigma }_{\perp }/{\sigma }_{\parallel }={R}_{\square }{d}_{s}/{\rho }_{\perp }\approx 0.0005$. These values were used to draw the figures. Though the specific values relevant to multilayer graphene are used for drawing figures, the theoretical results might apply to the spreading resistance of other anisotropic materials such as conducting polymers, Bi${}_{2}$Te${}_{3}$ films, and composite materials^41–45^. We show that the effect of the parallel electrical conductivity on the spreading resistance is through the modification of the true area radius $a$ by the effective area radius $a\sqrt{{\sigma }_{\parallel }/{\sigma }_{\perp }}$ as shown in Eq. (7). For thin anisotropic conductors, further amendment is required for the normalized film thickness *h*/*a* by $(h/a)\sqrt{{\sigma }_{\parallel }/{\sigma }_{\perp }}$ as discussed above Eq. (15). These quantities are beneficial to the highly anisotropic film as a cover layer when the in-plane electrical conductivity of the film is high and the electrical conductivity of the base slab denoted by ${\rho }_{b}$ is low; the condition can be written as ${\sigma }_{b} < \sqrt{{\sigma }_{\parallel }{\sigma }_{\perp }}$. Under the condition, the contact resistance decreases by increasing the layer thickness of the anisotropic conductor; the results correlate well with experimental findings and corroborate the conclusion that the measurement of spreading resistance could be useful in discriminating graphene layers of different thickness^37^.

So far, the transmission line model has been used to obtain the sheet resistance^29,46–51^. For a rectangular contact area of width $L$ and $W$, the total resistance calculated from the transmission line model for the current flowing in the parallel layer and that extracted by the metal contact is given in terms of the sheet resistance (*R*_□_) and the specific contact resistivity (${\rho }_{c}$) as $(\sqrt{{R}_{\square }{\rho }_{c}}/w)\,\coth (L\sqrt{{R}_{\square }/{\rho }_{c}})$^52,53^. The boundary conditions are different from our model. If the sheet resistance can be obtained by applying the transmission line model, the vertical electrical conductivity (${\sigma }_{\perp }$) can be estimated from the spreading resistance using our theoretical results. In this sense, our model is complementary to the transmission line model.

## Appendix A: Evaluation of the correction to Eq. (13)

According to Eq. (8), the electrostatic potential inside the cover layer can be expressed as

20 $${V}_{a}(r,z)={\int }_{0}^{\infty }\,\frac{dk}{k}{J}_{0}(kr)[{C}_{1}^{(A)}(k)\,\exp \,(kz\sqrt{{\sigma }_{\parallel }/{\sigma }_{\perp }})-{C}_{2}^{(A)}(k)\,\exp \,(-kz\sqrt{{\sigma }_{\parallel }/{\sigma }_{\perp }})],$$

and

21 $${E}_{z}(r,z)=-\,{\int }_{0}^{\infty }\,dk{J}_{0}(kr)[{C}_{1}^{(A)}(k)\,\exp \,(kz\sqrt{{\sigma }_{\parallel }/{\sigma }_{\perp }})+{C}_{2}^{(A)}(k)\,\exp \,(-kz\sqrt{{\sigma }_{\parallel }/{\sigma }_{\perp }})],$$

which is valid for $-\,h\ge z\ge 0$. The boundary conditions at the surface facing the circular contact are given by Eqs. (9) and (10). These boundary conditions can be expressed as the dual integral equations,

22 $${\int }_{0}^{\infty }\,\frac{dk}{k}[{C}_{1}^{(A)}(k)-{C}_{2}^{(A)}(k)]{J}_{0}(kr)={V}_{0}\,{\rm{for}}\,r\le a$$

23 $${\int }_{0}^{\infty }\,[{C}_{1}^{(A)}(k)+{C}_{2}^{(A)}(k)]{J}_{0}(kr)dk=0\,{\rm{for}}\,r > a.$$

To satisfy both boundary conditions, ${C}_{1}^{(A)}(k)-{C}_{2}^{(A)}(k)$ should satisfy

24 $${C}_{1}^{(A)}(k)-{C}_{2}^{(A)}(k)=\frac{2{V}_{0}}{\pi \,{\int }_{a}^{\infty }\,dtf(t)}\,{\int }_{a}^{\infty }\,dtf(t)\,\sin (kt),$$

where we determine *f*(*t*) below. By using^19,20,25,26^

25 $${\int }_{0}^{{\rm{\infty }}}\,\frac{dk}{k}\,\sin (kt){J}_{0}(kr)=\pi /2\,{\rm{i}}{\rm{f}}\,0\le r\le t\,{\rm{a}}{\rm{n}}{\rm{d}}\,0 < t$$

and noting that $r\le a\le t$, we show that Eq. (22) is satisfied. In other words, ${C}_{1}^{(A)}(k)-{C}_{2}^{(A)}(k)$ defined by Eq. (24) satisfies one of the boundary conditions given by Eq. (22). We define *B*(*k*) as

26 $$B(k)=\frac{{C}_{1}^{(A)}(k)+{C}_{2}^{(A)}(k)}{{C}_{1}^{(A)}(k)-{C}_{2}^{(A)}(k)}$$

and rewritten ${C}_{1}^{(A)}(k)+{C}_{2}^{(A)}(k)$ using Eq. (24) as

27 $${C}_{1}^{(A)}(k)+{C}_{2}^{(A)}(k)=\frac{2{V}_{0}}{\pi \,{\int }_{a}^{\infty }\,dtf(t)}B(k)\,{\int }_{a}^{\infty }\,dtf(t)\,\sin (kt).$$

By substituting Eq. (27), the other boundary condition given by Eq. (23) can be rewritten as

28 $$\frac{2}{\pi }\,{\int }_{0}^{\infty }\,dk\,{\int }_{a}^{\infty }\,dt\,{J}_{0}(kr)B(k)f(t)\,\sin (kt)=0\,{\rm{for}}\,r > a.$$

We first note

29 $$-\,\frac{{\rm{\partial }}}{{\rm{\partial }}r}\,{\int }_{r}^{{\rm{\infty }}}\,d\eta \frac{\eta {J}_{0}(k\eta )}{\sqrt{{\eta }^{2}-{r}^{2}}}=\,\sin (kr)$$

obtained from

30 $${\int }_{r}^{\infty }\,d\eta \frac{\eta {J}_{0}(k\eta )}{\sqrt{{\eta }^{2}-{r}^{2}}}=\,\cos (kr)/k.$$

The boundary condition given by Eq. (28) can be rewritten using Eq. (30) as

31 $$-\,\frac{2}{\pi }\frac{{\rm{\partial }}}{{\rm{\partial }}r}\,{\int }_{r}^{{\rm{\infty }}}\,d\eta \,\frac{\eta }{\sqrt{{\eta }^{2}-{r}^{2}}}\,{\int }_{0}^{{\rm{\infty }}}\,dk\,{\int }_{a}^{{\rm{\infty }}}\,dtf(t)\,\sin (kt){J}_{0}(k\eta )B(k)=0\,{\rm{f}}{\rm{o}}{\rm{r}}\,r > a,$$

which is satisfied when

32 $$\frac{2}{\pi }\,{\int }_{0}^{\infty }\,dk\,{\int }_{a}^{\infty }\,dtf(t)\,\sin (kt)\,\sin (kr)B(k)=0\,{\rm{for}}\,r > a$$

holds.

From the equipotential boundary condition at the bottom surface given by $V(r,-\,h)=0$, we obtain

33 $${C}_{2}^{(A)}(k)={C}_{1}^{(A)}(k)\,\exp \,(\,-\,2kh\sqrt{{\sigma }_{\parallel }/{\sigma }_{\perp }})$$

using Eq. (20). By substituting Eq. (33) into Eq. (24), we obtain ${C}_{1}^{(A)}(k)$. Using ${C}_{1}^{(A)}(k)$ and ${C}_{2}^{(A)}(k)$ thus determined, *B*(*k*) in Eq. (26) can be expressed as

34 $$B(k)=\,\coth (kh\sqrt{{\sigma }_{\parallel }/{\sigma }_{\perp }}).$$

The lowest order expression of the contact resistance for finite *h* is obtained by substituting ${f}_{\infty }(t)={c}_{\infty }\delta (t-a)$ into Eq. (24) and the result is given by Eq. (13). *c*_∞_ can be arbitrary set because of the normalization factor in Eq. (24). For convenience, we chose *c*_∞_ to satisfy ${c}_{\infty }\,{\int }_{a}^{\infty }\,dt\delta (t-a)=1$. The result satisfies the boundary condition given by Eq. (32) in the limit of $h\to \infty$; in the limit of $h\to \infty$, we have $\coth (kh)\to 1$ and Eq. (32) is confirmed by using

35 $$\frac{2}{\pi }\,{\int }_{0}^{\infty }\,dk\,\sin (kt)\,\sin (kr)=\delta (t-r)$$

and ${f}_{\infty }(t)=0$ for $t > a$ according to the definition of the delta function.

The correction term for finite *h* can be studied by introducing $f(r)={f}_{\infty }(r)+\delta f(r)$ and evaluate $\delta f(r)$. Equation (32) can be rewritten as

36 $$\delta f(r)-\frac{2}{\pi }\,{\int }_{0}^{\infty }\,d{k}_{1}\,{\int }_{a}^{\infty }\,dtf(t)\,\sin ({k}_{1}t)\,\sin ({k}_{1}r)[1-B({k}_{1})]=0\,{\rm{for}}\,r > a,$$

where we have used Eq. (35) and $f(r)=\delta f(r)$ for $r > a$ according to the definition of the delta function in ${f}_{\infty }(r)$. By applying sin(*kr*) on both sides of Eq. (36) and integrating over *r*, we obtain

37 $$\delta \hat{f}(k)-\frac{2}{\pi }\,{\int }_{a}^{\infty }\,dr\,\sin (kr)\,{\int }_{0}^{\infty }\,d{k}_{1}\,\hat{f}({k}_{1})\,\sin ({k}_{1}r)[1-B({k}_{1})]=0,$$

where we defined

38 $$\hat{f}(k)={\int }_{a}^{\infty }\,dr\,\sin (kr)f(r)\,{\rm{and}}\,\delta \hat{f}(k)={\int }_{a}^{\infty }\,dr\,\sin (kr)\delta f(r).$$

The double integral in Eq. (37) can be simplified by introducing

$$\begin{array}{rcl}{\int }_{a}^{\infty }\,dr\,\sin (kr)\,\sin ({k}_{1}r) & = & {\int }_{0}^{\infty }\,dr\,\sin (kr)\,\sin ({k}_{1}r)-{\int }_{0}^{a}\,dr\,\sin (kr)\,\sin ({k}_{1}r)\\  & = & \frac{\pi }{2}\delta (k-{k}_{1})-\frac{{k}_{1}\,\cos ({k}_{1}a)\,\sin (ka)-k\,\cos (ka)\,\sin ({k}_{1}a)}{{k}^{2}-{k}_{1}^{2}}.\end{array}$$

As a result, the integral equation is expressed by a single integral,

39 $$\begin{array}{c}\delta \hat{f}(k)B(k)+\frac{2}{\pi }\,{\int }_{0}^{{\rm{\infty }}}\,d{k}_{1}\,\hat{f}({k}_{1})[1-B({k}_{1})]\\ \,\times \frac{{k}_{1}\,\cos ({k}_{1}a)\,\sin (ka)-k\,\cos (ka)\,\sin ({k}_{1}a)}{{k}^{2}-{k}_{1}^{2}}=0.\end{array}$$

We note ${\mathrm{lim}}_{{k}_{1}\to k}[{k}_{1}\,\cos ({k}_{1})\,\sin (k)-k\,\cos (k)\,\sin ({k}_{1})]/({k}^{2}-{k}_{1}^{2})=0$. By substituting $\hat{f}({k}_{1})={\hat{f}}_{\infty }({k}_{1})+\delta \hat{f}({k}_{1})$, where ${\hat{f}}_{\infty }({k}_{1})={\int }_{a}^{\infty }\,dr\,\sin ({k}_{1}r){f}_{\infty }(r)=\,\sin ({k}_{1}a)$ into Eq. (39), we finally obtain

40 $$\delta \hat{f}(k)B(k)+\frac{2}{\pi }\,{\int }_{0}^{\infty }\,d{k}_{1}\,\delta \hat{f}({k}_{1})[1-B({k}_{1})]K(k,{k}_{1})={F}_{{\rm{r}}}(k),$$

where the right-hand side is given by

41 $${F}_{{\rm{r}}}(k)=\,\sin (ka)[1-B(k)]-\frac{2}{\pi }\,{\int }_{0}^{\infty }\,d{k}_{1}\,\sin ({k}_{1}a)[1-B({k}_{1})]K(k,{k}_{1}),$$

and the kernel is given by

42 $$K(k,{k}_{1})=\frac{{k}_{1}\,\cos ({k}_{1}a)\,\sin (ka)-k\,\cos (ka)\,\sin ({k}_{1}a)}{{k}^{2}-{k}_{1}^{2}}.$$

Equation (40) with Eqs. (41) and (42) is the Fredholm integral equation of the second kind which needs to be numerically solved.

The contact resistance is defined by $R=-\,{V}_{0}/{j}_{T}$, where *j*_*T*_ indicates the total current given by ${j}_{T}=2\pi \,{\int }_{0}^{a}\,rdr{j}_{z}(r,z=0)=2\pi {\sigma }_{\perp }\,{\int }_{0}^{a}\,rdr{E}_{z}(r,z=0)$; the current flowing through the circular disk in the z-direction obeys Ohm’s law given by

43 $${j}_{z}(r,z=0)={\sigma }_{\perp }{E}_{z}(r,z=0),$$

and the electric field is given by Eq. (21). The contact resistance can be expressed as

44 $$R={V}_{0}/\left[2\pi a{\sigma }_{\perp }\,{\int }_{0}^{\infty }\,\frac{dk}{k}[{C}_{1}(k)+{C}_{2}(k)]{J}_{1}(ka)\right],$$

where ${\int }_{0}^{a}\,dr\,r{J}_{0}(kr)=(a/k){J}_{1}(ka)$ is used. Using Eq. (27), the contact resistance can be expressed as

45 $$R=\left(1+\frac{2}{\pi }\,{\int }_{0}^{\infty }\,\frac{dk}{k}\,\cos (ka)\delta \hat{f}(k)\right)/\left(4a{\sigma }_{\perp }\,{\int }_{0}^{\infty }\,\frac{dk}{k}{J}_{1}(ka)B(k)\hat{f}(k)\right),$$

where we have introduced

46 $${\int }_{a}^{\infty }\,dt\,f(t)=1+(2/\pi )\,{\int }_{a}^{\infty }\,dt\,{\int }_{0}^{\infty }\,dk\,\sin (kt)\delta \hat{f}(k)$$

obtained from

47 $${\int }_{a}^{\infty }\,dt\,\delta f(t)=(2/\pi )\,{\int }_{a}^{\infty }\,dt\,{\int }_{0}^{\infty }\,dk\,\sin (kt)\delta \hat{f}(k)$$

and Eq. (35). The contact resistance can be evaluated by using $\hat{f}(k)=\,\sin (ka)+\delta \hat{f}(k)$, where $\delta \hat{f}(k)$ can be numerically calculated from Eqs. (40)–(42) by substituting *B*(*k*) defined by Eq. (34).

According to Eq. (40), $\delta \hat{f}(k)$ is an oscillating function whose frequency increases by increasing $k$. Rapidly oscillating $\delta \hat{f}(k)$ in Eq. (45) will give a negligible contribution to the value of the contact resistance. Numerical investigation of Eq. (40) indicates that the amplitude of $\delta \hat{f}(k)$ also decreases rapidly by increasing $k$ when $ka > 1$, where $\hat{f}(k)=\,\sin (ka)+\delta \hat{f}(k)$ is dominated by sin(*ka*). The largest deviation of $\hat{f}(k)$ from $\sin (ka)$ is obtained when $ka$ is around $1$. We truncate the upper-limit of the integration in Eq. (40) by 100/*a* and solve the equation by the standard discretization scheme. The results are shown in Fig. 3.

## Appendix B: Derivation of Eqs. (17) and (18) and the evaluation of the correction to these equations

The electrostatic potential inside the cover layer can be expressed as

48 $${V}_{a}(r,z)={\int }_{0}^{\infty }\,\frac{dk}{k}{J}_{0}(kr)[{C}_{1}^{(B)}(k)\,\exp \,(kz\sqrt{{\sigma }_{\parallel }/{\sigma }_{\perp }})-{C}_{2}^{(B)}(k)\,\exp \,(\,-\,kz\sqrt{{\sigma }_{\parallel }/{\sigma }_{\perp }})],$$

and the z-component of the electric field is obtained from ${E}_{z}(r,z)=-\,{\nabla }_{z}V(r,z)$ as

49 $${E}_{z}(r,z)=-\,{\int }_{0}^{\infty }\,dk{J}_{0}(kr)[{C}_{1}^{(B)}(k)\,\exp \,(kz\sqrt{{\sigma }_{\parallel }/{\sigma }_{\perp }})+{C}_{2}^{(B)}(k)\,\exp \,(\,-\,kz\sqrt{{\sigma }_{\parallel }/{\sigma }_{\perp }})],$$

which is valid for $-\,h\ge z\ge 0$. ${C}_{1}(k)$ and ${C}_{2}(k)$ are unknown constants to be determined from the boundary conditions. The electrostatic potential inside the base slab can be expressed as

50 $${V}_{b}(r,z)={\int }_{0}^{\infty }\,\frac{dk}{k}{J}_{0}(kr){C}_{3}(k)\,\exp \,(kz),$$

which is valid for $z\le -\,h$, and ${C}_{3}(k)$ is another unknown constant to be determined from the boundary conditions. Continuity of electrostatic potential can be expressed as^34,35^

51 $${V}_{a}(r,-\,h)={V}_{b}(r,-\,h).$$

Current continuity can be expressed as^34,35^

52 $${{\sigma }_{\perp }\frac{\partial {V}_{a}(r,z)}{\partial z}|}_{z=-h}={{\sigma }_{b}\frac{\partial {V}_{b}(r,z)}{\partial z}|}_{z=-h}.$$

The continuity conditions can be explicitly written as

53 $${C}_{1}^{(B)}(k)\,\exp \,(-kh\sqrt{{\sigma }_{\parallel }/{\sigma }_{\perp }})-{C}_{2}^{(B)}\,\exp \,(kh\sqrt{{\sigma }_{\parallel }/{\sigma }_{\perp }})={C}_{3}(k)\,\exp \,(-kh),$$

54 $${C}_{1}^{(B)}(k)\,\exp \,(-kh\sqrt{{\sigma }_{\parallel }/{\sigma }_{\perp }})+{C}_{2}^{(B)}\,\exp \,(kh\sqrt{{\sigma }_{\parallel }/{\sigma }_{\perp }})=\frac{{\sigma }_{b}}{\sqrt{{\sigma }_{\parallel }{\sigma }_{\perp }}}{C}_{3}(k)\,\exp \,(-kh).$$

Using the boundary condition for the electrostatic potential at the circular contact surface given by

55 $$(2{V}_{0}/\pi )\,{\int }_{0}^{\infty }\,\sin (ka){J}_{0}(kr)dk/k={V}_{0}\,{\rm{if}}\,r < a,$$

we also obtain

56 $${C}_{1}^{(B)}(k)-{C}_{2}^{(B)}(k)=\frac{2{V}_{0}}{\pi }\,\sin (ka).$$

Since we have 3 equations, Eqs. (53), (54) and (56), for 3 unknowns, ${C}_{1}^{(B)}(k)$, ${C}_{2}^{(B)}(k)$ and ${C}_{3}(k)$ can be determined. These are given by

57 $${C}_{1}^{(B)}(k)=\frac{{V}_{0}}{\pi }\frac{\sin (ka)\,\exp (kh\sqrt{{\sigma }_{\parallel }/{\sigma }_{\perp }})(1+{\sigma }_{b}/\,\sqrt{{\sigma }_{\parallel }{\sigma }_{\perp }})}{\cosh [kh\sqrt{{\sigma }_{\parallel }/{\sigma }_{\perp }}]+({\sigma }_{b}/\,\sqrt{{\sigma }_{\parallel }{\sigma }_{\perp }})\,\sinh [kh\sqrt{{\sigma }_{\parallel }/{\sigma }_{\perp }}]},$$

58 $${C}_{2}^{(B)}(k)=-\frac{{V}_{0}}{\pi }\frac{\sin (ka)\,\exp (-kh\sqrt{{\sigma }_{\parallel }/{\sigma }_{\perp }})(1-{\sigma }_{b}/\,\sqrt{{\sigma }_{\parallel }{\sigma }_{\perp }})}{\cosh [kh\sqrt{{\sigma }_{\parallel }/{\sigma }_{\perp }}]+({\sigma }_{b}/\sqrt{{\sigma }_{\parallel }{\sigma }_{\perp }})\,\sinh [kh\sqrt{{\sigma }_{\parallel }/{\sigma }_{\perp }}]},$$

59 $${C}_{3}(k)=\frac{{V}_{0}}{\pi }\frac{2\,\sin (ka)\,\exp \,(kh)}{\cosh \,[kh\sqrt{{\sigma }_{\parallel }/{\sigma }_{\perp }}]+({\sigma }_{b}/\sqrt{{\sigma }_{\parallel }{\sigma }_{\perp }})\,\sinh [kh\sqrt{{\sigma }_{\parallel }/{\sigma }_{\perp }}]}.$$

By substituting ${C}_{1}^{(B)}(k)$, ${C}_{2}^{(B)}(k)$ and ${C}_{3}(k)$ into Eq. (48), we find

60 $$\begin{array}{rcl}{V}_{a}(r,z) & = & \frac{2{V}_{0}}{\pi }\,{\int }_{0}^{\infty }\,\frac{dk}{k}\,\sin (ka){J}_{0}(kr)\\  &  & \times \,\frac{\cosh [k(z+h)\sqrt{{\sigma }_{\parallel }/{\sigma }_{\perp }}]+({\sigma }_{b}/\sqrt{{\sigma }_{\parallel }{\sigma }_{\perp }})\,\sinh [k(z+h)\sqrt{{\sigma }_{\parallel }/{\sigma }_{\perp }}]}{\cosh [kh\sqrt{{\sigma }_{\parallel }/{\sigma }_{\perp }}]+({\sigma }_{b}/\sqrt{{\sigma }_{\parallel }{\sigma }_{\perp }})\,\sinh [kh\sqrt{{\sigma }_{\parallel }/{\sigma }_{\perp }}]}.\end{array}$$

The electric field in the direction perpendicular to the circular contact surface is obtained from ${E}_{z}(r,z)=-\,{\nabla }_{z}{V}_{a}(r,z)$ as

61 $${E}_{z}(r,0)=-\,\frac{2{V}_{0}}{\pi }\sqrt{\frac{{\sigma }_{\parallel }}{{\sigma }_{\perp }}}\,{\int }_{0}^{\infty }\,dk\,\sin (ka){J}_{0}(kr)B(k),$$

where *B*(*k*) is given by

62 $$B(k)=\frac{\sinh [kh\sqrt{{\sigma }_{\parallel }/{\sigma }_{\perp }}]+({\sigma }_{b}/\sqrt{{\sigma }_{\parallel }{\sigma }_{\perp }})\,\cosh [kh\sqrt{{\sigma }_{\parallel }/{\sigma }_{\perp }}]}{\cosh [kh\sqrt{{\sigma }_{\parallel }/{\sigma }_{\perp }}]+({\sigma }_{b}/\sqrt{{\sigma }_{\parallel }{\sigma }_{\perp }})\,\sinh [kh\sqrt{{\sigma }_{\parallel }/{\sigma }_{\perp }}]}.$$

The current ${j}_{z}(r,z=0)$ obeys Ohm’s law given by Eq. (43) and the total current is given by ${j}_{T}=2\pi \,{\int }_{0}^{a}\,rdr{j}_{z}(r,z=0)$. The spreading resistance is obtained from $-\,{V}_{0}=R{j}_{T}$ as

63 $$R=\frac{1}{4a}\frac{1}{\sqrt{{\sigma }_{\perp }{\sigma }_{\parallel }}}\frac{1}{{C}_{F}},$$

and the correction factor is given by

64 $${C}_{F}={\int }_{0}^{\infty }\,\frac{d\lambda }{\lambda }\,\sin (\lambda ){J}_{1}(\lambda )B(\lambda /a).$$

A correction to Eq. (63) can be obtained from Eq. (45) using $\hat{f}(k)=\,\sin (ka)+\delta \hat{f}(k)$, where $\delta \hat{f}(k)$ can be numerically calculated from Eqs. (40)–(42) by substituting *B*(*k*) defined by Eq. (62). *B*(*k*) thus defined can be also found from Eq. (26) by substituting ${C}_{1}^{(B)}(k)$ and ${C}_{2}^{(B)}(k)$ for ${C}_{1}^{(A)}(k)$ and ${C}_{2}^{(A)}(k)$, respectively. This is the reason behind the use of Eqs. (40)–(42) with Eq. (62).
